# Insights into the Role of Circadian Rhythms in Bone Metabolism: A Promising Intervention Target?

**DOI:** 10.1155/2018/9156478

**Published:** 2018-09-27

**Authors:** Chao Song, Jia Wang, Brett Kim, Chanyi Lu, Zheng Zhang, Huiyong Liu, Honglei Kang, Yunlong Sun, Hanfeng Guan, Zhong Fang, Feng Li

**Affiliations:** ^1^Department of Orthopaedic Surgery, Tongji Hospital, Tongji Medical College, Huazhong University of Science and Technology, Wuhan, China; ^2^Department of Periodontics, School of Dental Medicine, University of Pennsylvania, Philadelphia, PA, USA; ^3^Biological Engineering and Regenerative Medicine Center, Tongji Hospital, Tongji Medical College, Huazhong University of Science and Technology, Wuhan, China

## Abstract

Numerous physiological processes of mammals, including bone metabolism, are regulated by the circadian clock system, which consists of a central regulator, the suprachiasmatic nucleus (SCN), and the peripheral oscillators of the BMAL1/CLOCK-PERs/CRYs system. Various bone turnover markers and bone metabolism-regulating hormones such as melatonin and parathyroid hormone (PTH) display diurnal rhythmicity. According to previous research, disruption of the circadian clock due to shift work, sleep restriction, or clock gene knockout is associated with osteoporosis or other abnormal bone metabolism, showing the importance of the circadian clock system for maintaining homeostasis of bone metabolism. Moreover, common causes of osteoporosis, including postmenopausal status and aging, are associated with changes in the circadian clock. In our previous research, we found that agonism of the circadian regulators REV-ERBs inhibits osteoclast differentiation and ameliorates ovariectomy-induced bone loss in mice, suggesting that clock genes may be promising intervention targets for abnormal bone metabolism. Moreover, osteoporosis interventions at different time points can provide varying degrees of bone protection, showing the importance of accounting for circadian rhythms for optimal curative effects in clinical treatment of osteoporosis. In this review, we summarize current knowledge about circadian rhythms and bone metabolism.

## 1. Introduction

Mammals have developed endogenous circadian clocks in response to Earth's rotation, characterized by approximately 24-hour cycles. Circadian clocks are maintained by a central regulator, the suprachiasmatic nucleus (SCN), located in the hypothalamus, as well as a peripheral circadian clock system consisting of BMAL1/CLOCK, PERs/CRYs, REV-ERBs/RORs, DECs, and DBP (molecular oscillators). Lesions on the SCN led to disrupted circadian rhythms in terms of temperature, sleep, and locomotor activity, and SCN transplantation restored circadian rhythms in SCN-ablated animals [1, 2]. Similar abnormal circadian rhythms were also observed in clock gene knockout mice [3].

As the master oscillator, SCN organizes the clocks in peripheral tissues through sympathetic pathways and release of glucocorticoids. Heart rate and plasma glucose content are regulated by SCN via the sympathetic nervous system [4, 5]. Moreover, glucocorticoids modulated by the master pacemaker synchronize peripheral circadian rhythms, acting as signals [6, 7]. Entrainment factors such as light and food synchronize the master circadian clock in the SCN. Light information reaches the SCN via the retina and the retinal-hypothalamic tract and serves as the most potent synchronizer. In contrast, food has a much less synchronizing influence on the SCN, with the exception of foods with high palatability [8, 9].

**Figure 1:**
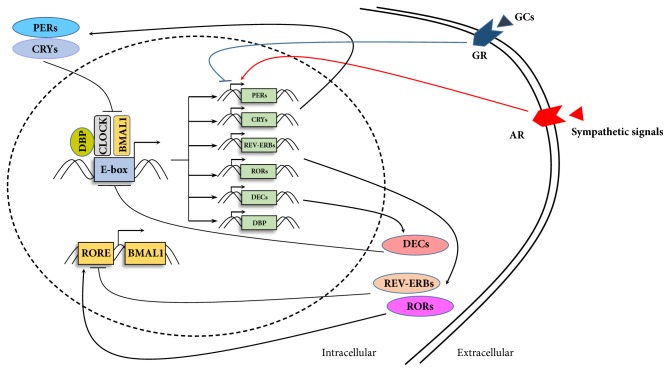
Molecular clock machinery. The feedback loop of clock genes and proteins is comprised of 2 main branches: CLOCK/BMAL1-PERs/CRYs and the supplementary regulators REV-ERBs, RORs, DECs and DBP. Glucocorticoids and sympathetic signals regulate circadian oscillators by modulating transactivation of PER1. CLOCK, circadian locomotor output cycles kaput; BMAL1, brain and muscle ARNT-like 1; PER, period circadian clock; CRY, cryptochrome; DBP, D-site binding protein; RORs, retinoid-related orphan receptors; RORE, ROR response element; DEC, differentiated embryo-chondrocyte expressed gene; GC, glucocorticoid; GR, glucocorticoid receptor; AR, adrenergic receptor.

In peripheral tissues, cellular circadian rhythms are maintained by molecular oscillators including BMAL1/CLOCK, PERs/CRYs, REV-ERBs/RORs, DECs and DBP. A complex including “circadian locomotor output cycles kaput” (CLOCK) and “brain and muscle ARNT-like 1” (BMAL1) drives transcription of target genes by binding to E-box elements in promoters, including those of periods (PER1, PER2, and PER3), cryptochromes (CRY1 and CRY2), reverse orientation c-erb *α* (REV-ERB*α*) and RAR-related orphan receptor *α* (ROR*α*). Heterodimers of PERs and CRYs, in turn, repress the transcriptional activities of BMAL1 and CLOCK [10]. Moreover, REV-ERB*α* suppresses transcription of BMAL1, whereas ROR*α* enhances it [11]. DEC1 and DEC2 repressed CLOCK/BMAL1-induced transactivation of the PER1 promoter by interacting with BMAL1 and competing for E-box elements [12]. BMAL1/CLOCK drives circadian transcription of DBP, which activates transcription of PER1 in conjunction with BMAL1/CLOCK [13, 14] (Figure 1).

Aging and postmenopausal status are the most common causes of primary osteoporosis. Ovariectomy attenuates oscillation of circadian clock genes in murine bones, including CLOCK, BMAL1, CRY2 and REV-ERB*α* [15]. Although aged mice exhibit normal oscillation amplitudes, peripheral clock responses to exercise and stress stimuli were impaired [16].

Our previous research found that REV-ERBs agonism suppressed RANKL-induced (receptor activator of nuclear factor kappa-Β ligand) osteoclast differentiation and ameliorated ovariectomy-induced bone loss [17]. In this review, we give an overview of correlations between bone metabolism and circadian rhythms in 6 parts: (1) circadian rhythms of bone metabolism; (2) impacts of circadian disruption on bone metabolism; (3) output signals of SCN regulate bone metabolism: sympathetic nervous signals and glucocorticoids; (4) hormones link circadian rhythms with bone metabolism; (5) Peripheral oscillators in bone; (6) implications of circadian rhythms in interventions for bone metabolism disorders.

## 2. Circadian Rhythm of Bone Metabolism

Bone metabolism exhibits daily patterns, represented by circadian rhythmicity, of bone turnover markers. In a study including 10 healthy men, the bone resorption marker C-terminal cross-linked telopeptide of type I collagen (CTX) displayed a diurnal rhythm, peaking at 05:30 (range 01:30 – 07:30) [18], which was consistent with previous studies [19, 20]. FGF23, a component of osteocyte function, also showed rhythmicity [21]. Similar rhythmicity was not found in sclerostin. Further, no rhythmic variations were observed in osteoprotegerin (OPG) or soluble RANKL (sRANKL) serum levels with a diurnal cycle [22, 23]. Paradoxically, another study showed that OPG displayed a circadian rhythm of a daytime increase and nocturnal decrease [24].

In dromedary camels, osteocalcin reaches its minimum and maximum concentrations of the 24-h period at 1300 h and at 1800 h, respectively [25]. In Gambian, Chinese, and white British adults, levels of plasma CTX, procollagen type 1 N-propeptide (P1NP), N-mid osteocalcin and bone alkaline phosphatase all show rhythmic patterns [26]. Intriguingly, the circadian rhythm of the CTX level can be broken by fasting but not by nasal administration of salmon calcitonin [19].

Using noninvasive Raman microscopy, McElderry et al. found that mineral deposition in calvarial tissue exhibits rhythmicity with a periodicity of 26.8±9.6 hours, with the earliest mineralization events occurring 6 hours after PER1 expression [27]. Similarly, the concentrations of calcium and inorganic phosphorus in rat metaphyseal bones displayed apparent circadian rhythms with peaks during dark periods [28].

In transgenic mice harboring the human osteocalcin promoter linked to a luciferase reporter gene, the maxillomandibular complex, calvaria, tail, carpals, and tarsals all displayed oscillatory patterns of osteogenic activity [29]. Moreover, Zvonic et al. showed that both elements of the circadian transcriptional apparatus (BMAL1/CLOCK/CRYs/PERs) and their mediators (DBP and REV-ERBs) exhibited oscillatory expression profiles in murine calvarial bones [30]. Russell et al. found that bone collagen formation also showed circadian fluctuations. Specifically, during assembly in dark periods, secretion of bone collagenous protein was decreased, as compared to light periods [31].

## 3. Impact of Circadian Disruption on Bone Metabolism

Sleep and eating are regulated by the circadian clock and can entrain circadian rhythms [32, 33]. Shift work, sleep restriction and fasting are common situations that may lead to circadian misalignments.

Shift workers are reported to have lower bone mineral density (BMD) and increased fracture risk compared to daytime workers [34, 35]. Deficit of 25-OH vitamin D3 caused by decreased open-air exposure may be responsible for this phenomenon [36]. In postmenopausal women, inadequate nocturnal sleep was associated with greater risk of bone loss [37]. Moreover, Swanson et al. reported that 3 weeks of circadian disruption with concurrent sleep restriction led to decreased P1NP and unchanged CTX [38]. A prospective cohort study demonstrated that the overall circadian rhythmicity of rest and activity (pseudo F-statistic) and daytime to nighttime activity ratio (alpha statistic) are associated with areal BMD (aBMD) of the total hip and femoral neck in older men [39]. Intriguingly, exposure to continuous light for 24 weeks is associated with 70% less rhythmicity of the SCN and with early osteoporosis in mice [40].

Eating times can alter the circadian rhythms of DNA synthesis and collagen synthesis in rat tibia [41]. Moreover, food administration leads to a peak of bone resorption. Dividing food into smaller portions blunted this effect and decreased bone resorption in thyroparathyroidectomized animals [42]. Urinary CrossLaps corrected with creatinine (U-CL/Cr), a bone resorption marker, exhibited a significant circadian rhythm, and its fluctuation was dampened by fasting [43].

## 4. Output Signals of SCN Regulate Bone Metabolism

**Figure 2:**
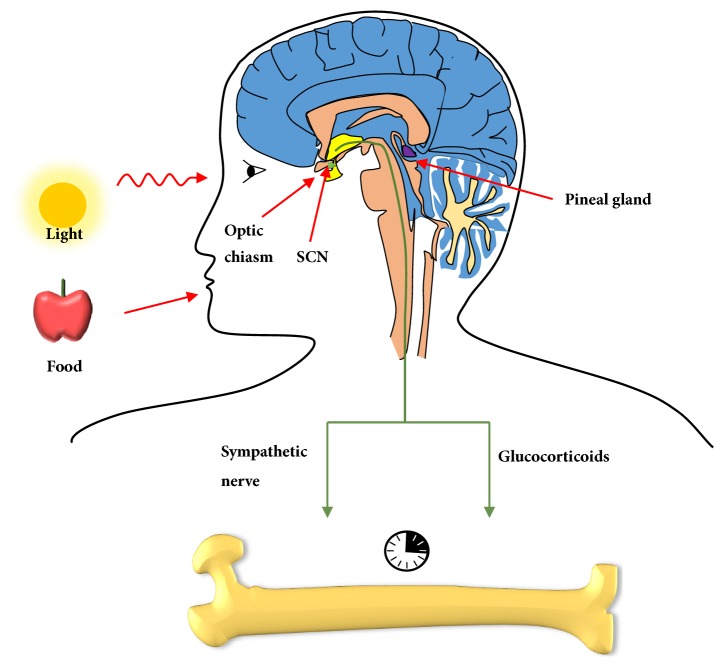
Role of the central pacemaker in bone metabolism. Light (major entrainment factor) and food signals entrain circadian rhythms in SCN, which transmits circadian signals to bones through hormones and sympathetic nervous functions.

Circadian signals are transmitted from the central pacemaker SCN to peripheral tissues through the sympathetic nervous system and glucocorticoids [7, 44, 45] (Figure 2).* In vivo* administration of adrenaline or noradrenaline increased expression of PER1 in the murine liver. Daily injection of adrenaline recovered oscillation of PER1 and PER2 expression in the livers of mice with disrupted SCNs [46]. Glucocorticoids (cortisol in primates and corticosterone in rodents) secreted by adrenocortical steroidogenic cells exhibit circadian rhythms and are involved in regulation of lipid metabolism, glucose homeostasis and cardiovascular tone [47]. SCN lesions in rats altered synchronization of the adrenal corticosterone rhythm [48]. After glucocorticoids bind to glucocorticoid receptors (GRs), these GRs regulate PER1 directly, rather than through PER2 expression, by binding to a glucocorticoid-responsive element [49].

### 4.1. Sympathetic Nervous Functions

Treatment with *β*-adrenergic receptor agonist (isoprenaline, Iso) or a synthetic glucocorticoid (dexamethasone) immediately induces human osteoblast SaM-1 cells to express hPER1 and hPER2 and causes circadian oscillation of these two genes, with three peaks observed within 48 h [50]. Moreover, Iso induces rhythmic expression of the osteoblast-related gene Col1a1. In MC3T3-E1 osteoblastic cells, Iso caused oscillations of PER2 expression [51]. Exposure to phenylephrine (PHE), a nonspecific *α*1-adrenoreceptor (AR) agonist, induced rhythmic expression of bone morphogenetic protein 4 (BMP4) in MC3T3-E1 cells [52], which promotes osteoblast differentiation [53]. Systemic administration of prazosin, a nonspecific *α*1-AR antagonist, led to decreased bone formation [54]. In addition, *α*1 *β*-AR-deficient mice exhibited a lower bone mass compared with wild-type mice [54].

FGF23 is an important cytokine that regulates phosphate and vitamin D metabolism. Iso induced FGF23 expression in the femur, but this was not observed in BMAL1 knockout mice [21].

### 4.2. Glucocorticoids

SCN orchestrates the daily rhythms of circulating glucocorticoids in 2 ways: (1) regulating the hypothalamic-pituitary-adrenal (HPA) axis through efferent projection to paraventricular nucleus (PVN) [55]; (2) inducing gene expression in the adrenal gland via the sympathetic nervous system [45]. Both PER1 deficient PER1^Brd^ mice and PER2-/- mice exhibit impaired circadian rhythmicity of circulating glucocorticoids [56, 57]. Glucocorticoids further regulate expression of molecular oscillators through binding to glucocorticoid receptor (GR) in peripheral organs, including heart, liver and kidney [7]. Moreover, Wu X et al. revealed that treatment of murine and human bone marrow mesenchymal stem cells (MSC) with dexamethasone (a synthetic glucocorticoid) synchronizes rhythmic expression of BMAL1, PER3, REV-ERB*α* and REV-ERB*β* [58]. CLOCK/BMAL1 in turn interact with GR and suppress GR-induced transcriptional activity [59].

The impact of glucocorticoids on bone metabolism has been comprehensively investigated in the past decades. Physiological glucocorticoids signaling in osteoblast progenitors is essential for maintaining the normal bone density, whereas endogenous glucocorticoid excess and high dose therapeutic glucocorticoids result in osteoporosis by suppressing osteoblast activity and bone formation [55, 60].* In vitro* study demonstrated that a glucocorticoid rather than a *β*-agonist synchronized circadian expression of clock and osteoclast-related genes in osteoclasts [61]. Moreover, dexamethasone injection recovered circadian expression of cathepsin K (CTSK) and nuclear factor of activated T-cells, cytoplasmic 1 (NFATc1), in adrenalectomized mice [61].

## 5. Hormones Link Circadian Rhythms with Bone Metabolism

Aside from sympathetic and glucocorticoid signaling, hormones such as melatonin, ghrelin and parathyroid hormone (PTH) can also coordinate circadian rhythms in bone tissue.

### 5.1. Melatonin

Melatonin is a hormone that regulates sleep and other circadian activities [62]. The SCN regulates melatonin secretion through an inhibitory projection to the paraventricular nucleus, which controls sympathetic output to the pineal gland [63]. Several animal studies have demonstrated that melatonin has beneficial effects on bone metabolism, promoting osteoblast differentiation and suppressing osteoclast formation through oxidative stress reduction and PPAR*γ* suppression [64–66]. On the other hand, nocturnal melatonin administration did not improve bone parameters in a blind model using MMTV-*Neu *transgenic mice [67]. Moreover, a double-blind clinical study demonstrated that there was no difference in bone density, osteocalcin, or NTX between nightly melatonin supplementation and placebo groups [68].

### 5.2. Ghrelin

Levels of the orexigenic peptide ghrelin exhibit circadian rhythms, and this hormone has been found to induce a phase advance of circadian time in the SCN of about 3 h* in vitro* [69], causing expression of ghrelin receptor (GHSR). GHSR knockout mice displayed lengthening of their circadian period and greater activity in anticipation of a scheduled meal under constant lighting conditions [70]. In terms of bone metabolism, ghrelin has been shown to promote proliferation and prevent apoptosis of osteoblasts. However, clinical studies of serum ghrelin and BMD demonstrated no correlation between them [71, 72].

### 5.3. PTH

PTH exhibits a moderate increase between 16:00 and 19:00 and a broader, longer-lasting increase from late evening to early morning, reaching its peak between 02:00 and 06:00 [26, 73]. The direct connection between SCN and PTH secretion remains uncharacterized.

Constitutively active PTH receptors expressed in osteoblasts promote PER1 expression [74]. In organ-cultured murine femur, Okubo et al. revealed that PTH reset the circadian oscillation of PER2::luciferase activity in a time- and dose-dependent manner [75]. Moreover, PTH administration shifts the peak time of PER2::luciferase activity in fracture sites and growth plates [76]

## 6. Peripheral Oscillators in Bone

Circadian rhythms in peripheral tissues are governed by an autoregulatory transcriptional and translational feedback loop with two branches: the positive transcriptional branch comprised of CLOCK and BMAL1 and the negative branch formed by PERs and CRYs. Circadian regulation genes including REV-ERBs, RORs, DECs and DBP play vital roles in the circadian system. Among these genes, BMAL1 (ARNTL), DBP, NR1D1 (REV-ERB*α*), NR1D2 (REV-ERB*β*), PER1, PER2, and PER3 have been observed to oscillate in all mammalian organs, including the SCN [77, 78]. In human mesenchymal stem cells, expression of the circadian genes CLOCK, BMAL1, PER1, and PER2 displayed rhythmicity [79]. These clock genes play complex roles in bone metabolism (Table 1).

### 6.1. CLOCK

Homozygous CLOCK mutant mice (*Clock*^△19/△19^) were reported to display lengthened circadian periods (26-28h in length) [80]. On the other hand, Debruyne et al. reported that CLOCK knockout mice showed robust circadian rhythms in locomotor activity [81]. Subsequent research from the same group demonstrated that NPAS2 functionally substitutes for CLOCK in SCN [82]. In terms of bone metabolism, CLOCK mutant mice exhibit decreased bone density due to reduced expression of Pdia3, a 1, 25-dihydroxy-vitamin D3 receptor [83]. Pdia3 deficiency resulted in reduced relative bone volume (BV/TV) and trabecular number [84].

### 6.2. BMAL1

Mice deficient in BMAL1 displayed impaired locomotor activity during light-dark (LD) cycles as well as various symptoms of premature aging, including sarcopenia, cataracts, reduced subcutaneous fat and organ shrinkage [85, 86]. Deletion of Bmal1 results in low bone mass [87]. Conditional deletion of Bmal1 in osteoblasts led to reduced bone loss and increased bone resorption. Subsequent coculturing revealed that Bmal1-deficient osteoblasts promote osteoclastogenesis [88].

### 6.3. PERs

Both PER1- and PER2-deficient mice showed severely disrupted locomotor activity rhythms [89]. Light-induced expression of PER1 and PER2 proteins in the SCN is essential for entrainment [90].

According to Fu et al., mice lacking PER1, PER2 or the PER2 PAS domain (PER2^m/m^) exhibit normal bone volume, whereas PER1^−/−^; PER2^−/−^ and PER1^−/−^; PER2^m/m^ mice display increased bone mass. Leptin intracerebroventricular infusion further increases bone mass of PER1^−/−^; PER2^m/m^ mice [91].

In contrast, Maronde et al. reported that PER2-mutant PER2^Brdm1^ mice displayed greater bone volumes compared with wild-type mice [92].

### 6.4. CRYs

The circadian period is 1 hour shorter in CRY1 knockout mice, whereas it is 1 hour longer in CRY2 knockout mice [93–95]. CRY2-deficient mice display increased bone volume with low osteoclast activity [94]. Moreover, mice lacking CRY1 and CRY2 exhibit increased bone volume [91]. In a Chinese pediatric cohort, Cry2 rs2292910 was associated with osteoporosis (r = -0.082, p = 0.045) [96].

### 6.5. REV-ERBs

REV-ERB*α* and REV-ERB*β* were found to regulate hepatic lipid metabolism and skeletal muscle activity in addition to their roles in the cell autonomous clock [97, 98].

Our previous work demonstrated that REV-ERB agonism of SR9009 suppresses osteoclast formation and ameliorates ovariectomy-induced bone loss [17]. REV-ERB*α* was significantly increased in bone mesenchymal stem cells of old rhesus monkeys, which were characterized by decreased osteogenetic capacity and significantly increased REV-ERB*α* expression [99]. Furthermore, overexpression of REV-ERB*α* in bone mesenchymal stem cells inhibited osteogenesis [100].

### 6.6. RORs

RORs regulate target gene expression by binding to ROR response elements (RORE) containing the RGGTCA consensus motif [101]. ROR*α* (sg/sg) mice displayed a shortened period of the locomotor activity rhythm, while ROR*β* knockout mice showed a lengthened circadian period in constant dark [102, 103].

ROR*α* (sg/sg) mice with a deletion within the ROR*α* gene are osteopenic compared with wild-type and heterozygote littermates [104], indicating the essential role of ROR*α* in regulation of normal bone metabolism. Further investigation demonstrates that ROR*α* directly activates bone sialoprotein expression, which promotes osteoblast differentiation and matrix mineralization [105].

Expression of ROR*β* is increased in bone marrow precursor cells of aged mice and bone biopsies from postmenopausal women [106]. Overexpression of ROR*β* in MC3T3-E1 cells results in decreased bone nodule formation [107]. Knockout of the ROR*β* gene in osteoblasts promotes expression of osteogenic genes and osteoprotegerin (OPG). Consistent with* in vitro* results, ROR*β*-/- mice exhibited increased bone volume [108].

### 6.7. DECs

DEC1 overexpression extended the periods of clock genes such as DEC1, DEC2 and PER1 [109]. Longer circadian periods under conditions of constant darkness were observed in DEC1 knockout mice [109]. However, DEC2 knockout mice showed no changes in their circadian activity rhythms [110].

After osteogenic induction of MSC in medium containing dexamethasone, beta-glycerophosphate, and ascorbic acid, DEC1 expression gradually increased from day 5 to day 14 [111]. Overexpression of DEC1 in growth plate chondrocytes at the prehypertrophic stage increased mRNA levels of Indian hedgehog, Runx2, and type X collagen, and also promoted alkaline phosphatase activity and mineralization [112].

### 6.8. DBP

DBP, also known as group-specific component (GC), is a polymorphic protein that binds 85-90% of the total circulating 25-OH vitamin D, which is later transported to the kidneys [113]. In the kidneys, 25-OH vitamin D is converted to the active form of vitamin D, 1,25-dihydroxy-vitamin D (1,25(OH)_2_D). DBP can also be converted to DBP-macrophage activating factor (DBP-MAF), which directly promotes osteoclast formation [114]. DBP protein and mRNA in the SCN and various peripheral tissues display strong circadian rhythmicity [115]. DBP activates transcription of PER1 in cooperation with CLOCK/BMAL1, which regulate the circadian transcription of DBP through interaction with E boxes [14].

Genotyping based on rs4588 of the GC gene showed that total hip-bone mineral content was associated with GC genotype (p* *=* *0.05, ANCOVA) in boys [116]. In women of postmenopausal age, DBP*∗*10 (with 10 repeats of TAAA) is associated with lower incidence of bone fracture compared with relevant controls, while DBP*∗*11 is related to higher incidence [117]. Another study showed that DBP*∗*10 and DBP*∗*11 are both associated with lower risks of osteoporosis [118]. A highly suggestive association was found between DBP SNPs (single nucleotide polymorphisms) and spine BMD [119].

Despite associations between DBP genotype and BMD, DBP-deficient mice displayed normal bone volume [120].

## 7. Implications of Circadian Rhythms in Interventions for Bone Metabolism Disorders

Salmon calcitonin (sCT) is a widely used antiosteoporosis drug. Karsdal et al. assessed CTX-I levels after oral salmon calcitonin intake at three different time points (morning, pre-dinner and evening) in healthy postmenopausal women and found that pre-dinner dosing of oral sCT resulted in optimal efficacy of CTX-I downregulation [125].

PTH is the only FDA approved anabolic drug for osteoporosis. A 12-month clinical investigation demonstrated that morning administration of teriparatide resulted in higher spine BMD compared to evening application [126].

Pulsed electromagnetic fields (PEMF) have been reported as an effective method of preventing osteoporosis, and daytime PEMF application from 9:00 to 15:00 was more effective for prevention of ovariectomy-induced bone loss than nighttime PEMF from 0:00 to 6:00 [127].

Together, these results indicate that circadian rhythms should be considered when treating bone disorders.

## 8. Conclusions

Bones are organs under dynamic regulation of osteoblasts, osteoclasts, osteocytes, and bone lining cells. Disruption of the balance between these cells leads to diseases such as osteoporosis. The prevalence of osteoporosis and low bone mass is estimated at 10.3% and 43.9% among US adults aged 50 and older, respectively [128]. According to a 2002 national population sample, the annual cost of osteoporosis and fractures in elderly patients in the US was up to $16 billion [129].

Circadian rhythms are regulated by the central oscillator SCN and the peripheral clock system, which contribute to maintaining homeostasis of physiological functions. Circadian arrhythmia is related to disorders such as neurodegenerative diseases [130], metabolic syndrome, cancer [131], and cardiovascular disease [132]. Increasing evidence demonstrates that the circadian clock systems, including the master pacemaker and peripheral circadian machinery, play pivotal roles in maintaining homeostasis of bone metabolism. Circadian disruption due to sleep restriction, shift work, fasting, or knockout of clock genes results in disrupted homeostasis of bone metabolism.

Among the various molecular oscillators, REV-ERBs and RORs are nuclear receptors that can be activated by their natural ligands, heme, and oxygenated sterols, respectively [133]. Various synthetic ligands with high affinities for REV-ERBs and RORs have been developed based on their structures. These ligands display promising therapeutic effects in animal studies, including those on tumors [134], type 1 diabetes [135], atherosclerosis [136], and autism [137]. Combining these studies with our previous findings [17], REV-ERBs and RORs appear to offer promising therapy targets for several disorders, including osteoporosis [138].

In this review, we provide insight into the role of circadian rhythms in bone metabolism, and show evidence that the circadian system may be a promising target of clinical intervention for abnormal bone metabolism.

## Figures and Tables

**Table 1:** Summary of circadian and bone phenotypes in clock gene transgenic mice.

Gene	Circadian phenotype	Bone phenotype
CLOCK	CLOCK mutant mice (*Clock*^△19/△19^): long circadian period (26-28h) [80] CLOCK knockout mice: robust circadian rhythm in locomotor activity [81]	Clock mutant mice: low bone mass [83]

BMAL1	BMAL1-/- mice: impaired locomotor activity [87]	BMAL1 knockout mice: low bone mass [87]

PERs	PER1-deficient mice: disrupted circadian behavioral rhythms [89] PER2-deficient mice: disrupted circadian behavioral rhythms [89]	PER1-deficient mice: normal bone volume [91] PER2-deficient mice: normal bone volume [91] PER2 PAS domain (PER2^m/m^): normal bone volume [91] PER1^−/−^;PER2^m/m^: significantly increased bone mass [91] PER1-/-; PER2-/-: increased bone mass [91] Per2^Brdm1^ mice: increased bone volume at 3, 12 and 48 weeks [92]

CRYs	CRY1-deficient mice: circadian period 1 hour shorter than wild type [93, 94] CRY2-deficient mice: circadian period 1 hour longer than wild type [95] CRY1/2 double knockout mice: arrhythmic in constant darkness [121]	CRY1-deficient mice: not reported CRY2-deficient mice: increased bone volume [94] CRY1/2 double knockout mice: high bone volume [91]

REV-ERBs	REV-ERB*α* knockout: average period length significantly shorter [122] REV-ERB*β* knockout: no changes in circadian activity rhythms [123] REV-ERB*α*/*β* double knockout: the free running period length in constant darkness was 2.5 hours shorter [123]	REV-ERB*α* knockout: not reported REV-ERB*β* knockout: not reported REV-ERB*α*/*β* agonist SR9009: suppressed osteoclast formation and ameliorated OVX-induced bone loss [17]

RORs	ROR*α* (sg/sg): shortened period length of the locomotor activity rhythm [102] ROR*β* knockout: significant increase in circadian period in constant dark [103]	ROR*α* (*sg/sg*): osteopenic [104] ROR*β* knockout: increase bone mass [108]

DECs	DEC1 knockout: longer circadian period under conditions of constant darkness [109] DEC2 knockout: no changes in circadian activity rhythms [110]	DEC1 or DEC2 knockout: not reported

DBP	DBP knockout: shorter period [124]	DBP knockout: normal bone volume [120]
